# Nucleotide diversity analysis highlights functionally important genomic regions

**DOI:** 10.1038/srep35730

**Published:** 2016-10-24

**Authors:** Tatiana V. Tatarinova, Evgeny Chekalin, Yuri Nikolsky, Sergey Bruskin, Dmitry Chebotarov, Kenneth L. McNally, Nickolai Alexandrov

**Affiliations:** 1 Center for Personalized Medicine and Spatial Sciences Institute, University of Southern California, Los Angeles, CA, USA; 2 Kharkevich Institute for Information Transmission Problems, Russian Academy of Sciences, Moscow, Russian Federation; 3 Vavilov Institute of General Genetics, Moscow, Russia; 4 F1 Genomics, San Diego, CA, USA; 5 School of Systems Biology, George Mason University, VA, USA; 6 International Rice Research Institute, Los Baños, Laguna 4031, Philippines

## Abstract

We analyzed functionality and relative distribution of genetic variants across the complete *Oryza sativa* genome, using the 40 million single nucleotide polymorphisms (SNPs) dataset from the 3,000 Rice Genomes Project (http://snp-seek.irri.org), the largest and highest density SNP collection for any higher plant. We have shown that the DNA-binding transcription factors (TFs) are the most conserved group of genes, whereas kinases and membrane-localized transporters are the most variable ones. TFs may be conserved because they belong to some of the most connected regulatory hubs that modulate transcription of vast downstream gene networks, whereas signaling kinases and transporters need to adapt rapidly to changing environmental conditions. In general, the observed profound patterns of nucleotide variability reveal functionally important genomic regions. As expected, nucleotide diversity is much higher in intergenic regions than within gene bodies (regions spanning gene models), and protein-coding sequences are more conserved than untranslated gene regions. We have observed a sharp decline in nucleotide diversity that begins at about 250 nucleotides upstream of the transcription start and reaches minimal diversity exactly at the transcription start. We found the transcription termination sites to have remarkably symmetrical patterns of SNP density, implying presence of functional sites near transcription termination. Also, nucleotide diversity was significantly lower near 3′ UTRs, the area rich with regulatory regions.

Understanding the relationship between genotype and phenotype is a key issue in life sciences with hugely important implications in biomedical R&D, healthcare and agriculture. Innate genetic variability is both the source and consequence of selection in populations of humans, crops and animals. There is a fine balance between the variability in DNA sequences and the evolutionary constraints for conservation of the original state or fixation of a new variant with a selective advantage. Most genetic variants occur as single nucleotide polymorphisms (SNPs) and small insertions and deletions (indels). In eukaryotic genomes, distribution of variants and the rate of spontaneous mutations are not uniform across individual sites^1^,^2^. Instead, they depend on functional impact of variants on either the protein structure or the RNA structure in the regulatory regions affecting transcription. Thus, a SNP causing a premature stop codon will truncate a protein sequence, which may be phenotypically significant. Not surprisingly, genetic variability in protein coding regions is two to three times lower than in intergenic regions^2^,^3^,^4^,^5^. Similarly, promoter regions (and especially the sequences within transcription factor binding sites, TFBS) are less prone to SNPs and indels than intergenic regions, with the levels of sequence variation within and around TFBSs inferred from their position weight matrix^6^. Genetic variability can be also assessed from an evolutionary point of view, using a combination of phylogenetic and population genetic techniques^7^. Thus, mutational “hot” and “cold” spots were discovered by analysis of a population of 34 *E. coli* strains^7^. A lower mutation rate was observed in highly expressed genes and in those undergoing stronger purifying selection that reduces retention of deleterious mutations^7^. In another publication, mutational hotspots were linked to the regions of open chromatin in mammalian genomes^8^. Both in normal and cancerous tissues, mutation rates were found to be related to epigenetic features, such as DNA methylation and nucleosome occupancy^9^,^10^, with disproportionately higher numbers of SNPs occurring in variably methylated DNA regions^11^. Nucleotide composition was also found to be an important factor in SNP distribution^12^,^13^,^14^,^15^, with a quadratic dependence of SNP density on GC content^12^,^13^. Methylation of cytosines in high-GC regions results in locally increased SNP density^16^. High SNP density is also characteristic for low-GC regions, typically packed into heterochromatin and frequently referred to as “gene deserts”^8^. On the finer scale, 5′ and 3′ untranslated regions of protein-encoding genes (UTRs) feature conserved regions, as revealed by analysis of patterns of sequence conservation in yeast and mammals^17^. In some cases, SNP density in these regions is even lower than in the corresponding coding regions^18^. Conserved areas of UTRs contain binding sites for proteins or antisense RNAs that modulate transport, RNA stability, cellular localization, expression level and translation^17^. Finally, the same nucleotide positions tend to be conserved both between organisms and within organisms, as was established by cross-species analysis of SNP distribution in functionally important regions in mice and men^19^.

Most studies on genomic variability have been conducted on mammals and yeast^17^,^18^,^19^, with much lesser attention to plants^20^,^21^,^22^,^23^,^24^,^25^. Analysis of genomic variation in rice was recently presented by McCouch *et al*.^26^ (1,568 diverse inbred rice varieties analyzed at 700,000 SNPs), Huang *et al*.^27^ (rice domestication analysis based on 1,083 cultivated indica and japonica varieties), Xu *et al*.^28^ (resequencing of 50 accessions at >15 × raw coverage), Duitama *et al*.^29^ (sequencing and bioinformatics analyses of 104 rice varieties belonging to the major subspecies of *Oryza sativa*), Arai-Kichise *et al*.^30^ (analysis of SNP in seven rice cultivars of temperate and tropical japonica), Jain *et al*.^2^ (whole-genome resequencing of three rice cultivars with contrasting responses to drought and salinity stress).

Understanding DNA variability in plants is hugely important, as crops are the core of world’s agriculture. Food consumption is predicted to double within the next 35 years, which translates in the demand for 2% annual yield growth rates for major crops. Yet, current breeding and selection methods, including transgenic technology, can only support on average a 1.1% annual growth rate^31^. Rice is the key cereal for the majority of the global population, especially in Asia and Africa. Climate change and increasing lack of agricultural land pose further challenges to rice production. Achieving the required increase of global production by up to 50% by 2050^32^ will require new technologies, such as genomic selection and genome editing. Genomic selection depends on selection of those SNPs that favorably contribute to phenotype. Generally, success of new computational methods relies on understanding the specifics of plant biology, not shared with animals, such as self-fertilization, polyploidy and higher genetic variability^33^,^34^,^35^. Also, dicot and monocot plants have their own unique genetic features, and, therefore, one cannot liberally extrapolate knowledge from one plant to another, for example from the model dicot *Arabidopsis thaliana* to grasses^36^,^37^,^38^.

Here, we present a large-scale study on genetic variability in the rice genome, based on high quality data from the SNP-seek database, with over 40 million SNPs from 3,000 rice genomes^25^, the largest source of plant SNPs to date. Accessing this unique database allowed us to precisely describe genome-wide patterns of variability and to detect pronounced patterns not seen before. We provide new evidence on close association between local genetic variability and functionality, and suggest that the patterns of SNP density can help to detect functionally important genomic regions that are essential for crop improvement. Thus, we discovered specific characteristic patterns near sites with well-defined biological functions, such as transcription and translation start and stop sites, promoters, intron/exon junctions and 5′ and 3′ UTRs. Some features are similar to those in animals, while the others are novel and unique. In addition, we calculated relative SNP density on different groups of genes and demonstrated that transcription factor-encoding genes are highly conserved, whereas kinases and membrane-bound transporters are the most variable gene families.

## Results

### Genome-wide mutation rates

We analyzed the distribution of SNPs in the genome of *Oryza sativa.* We found that the minor allele frequency (MAF) distribution varies between parts of genome. The original 29M dataset of bi-allelic SNPs mapped to the Nipponbare genome contains many rare SNPs: 22% of them have MAF < 0.00035 (occurring in one genome in 3,000 in a homozygous state), and 67% of them have MAF < 0.01 (present in less than 30 genomes). The genome-wide average MAF is 0.052 while the mean MAF for CDS is 0.028 and, contrastingly, 0.061 for introns. Exons contain more SNPs with lower MAF scores as compared to introns and UTRs. With an increase in the MAF cut-off (to control sequencing errors), a larger portion of SNPs in coding regions are excluded from the dataset. Rare exonic SNPs originated from five genomes of *Oryza glaberrima*, showing massive levels of genomic structural variation, with particularly high instability in defense-related genes^39^. These accessions were excluded from our analysis since they are too evolutionary distant from *O. sativa*. Twelve other genomes were excluded since one had a very low coverage and others had excessive numbers of heterozygous singleton SNPs, which is highly unlikely for an inbred species. To reduce the number of possible false positive polymorphisms, we imposed two levels of constraints, as described in Materials and Methods, arriving at two datasets (“Base” 16M, and “Filtered” 5M) for the analysis (Table 1).

### Transcription start and termination sites

**Figure 1:**
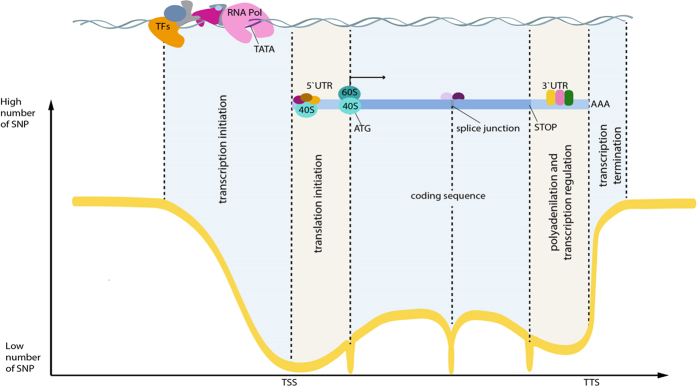
Regions near translation and transcription start and termination sites are more conserved than the surrounding genic and intergenic regions.

**Figure 2:**
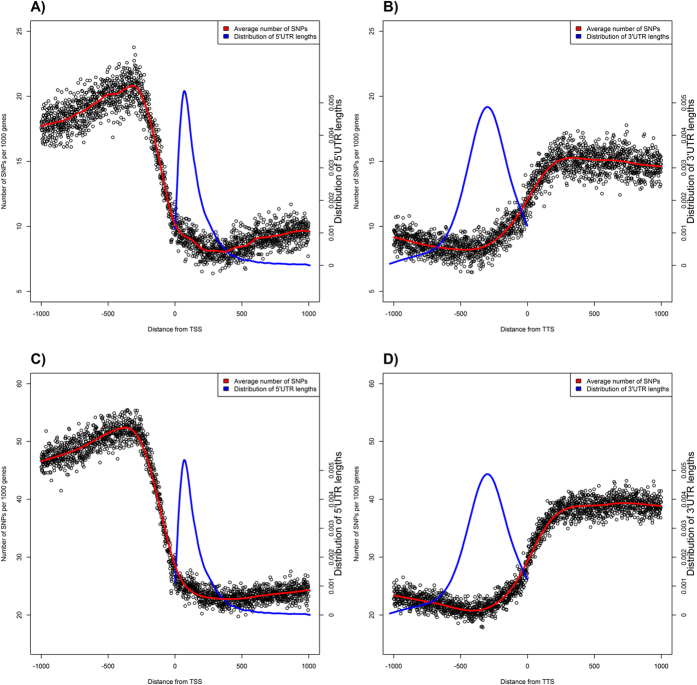
Density of SNP near the transcription start site (TSS), panels (**A**,**C**); and termination site (TTS), panels (**B**,**D**). Panels (**A**,**B**) correspond to the “Filtered” dataset, panels (**C**,**D**) to the “Base” dataset. TSS regions contain the following amount of SNPs: 553,800 “Filtered” and 1,443,188 “Base” datasets. TTS regions contain the following amount of SNPs: 482,685 “Filtered” and 1,231,570 “Base” datasets. Blue curve shows the distribution of 5′ (**A,C**) and 3′ (**B,D**) UTR lengths. Whole-genome fraction of variable positions is 1.3% for the “Filtered” and 4.4% for the “Base” datasets, respectively.

**Figure 3:**
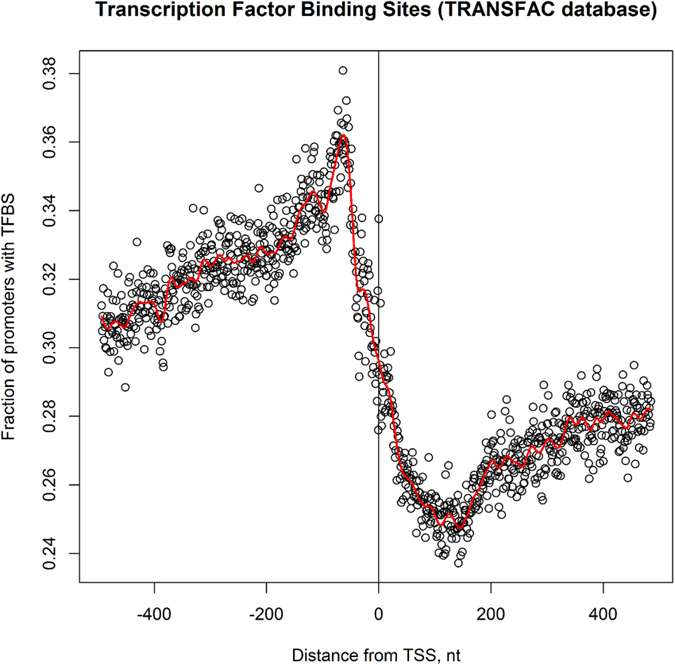
Distribution of TFBS in promoters of *O. sativa* calculated by TRANSFAC MATCH program with the minimum core similarity = 1.

**Figure 4:**
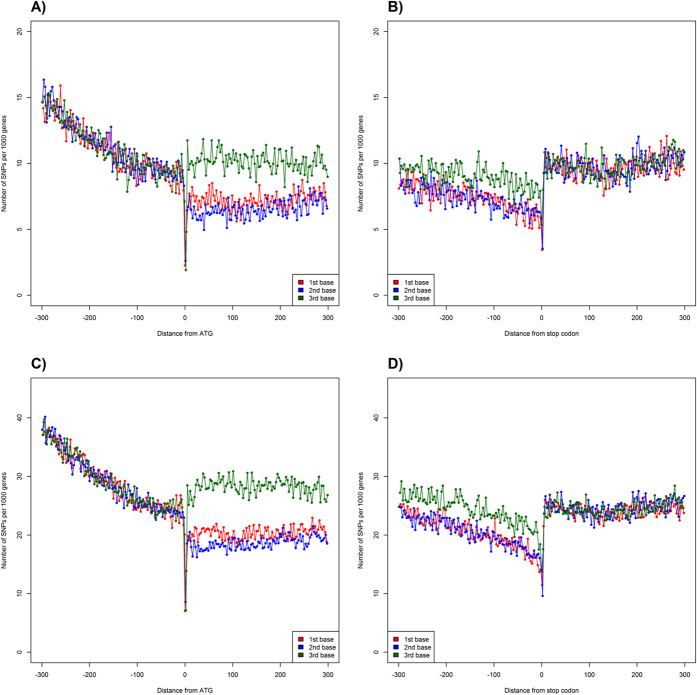
Density of SNP near the translation start site (ATG), panels (**A**,**C**); and termination site (TER) panels (**B**,**D**). Panels (**A**,**B**) correspond to the “Filtered” dataset, panels (**C**,**D**) to the “Base” dataset. ATG regions contain the following amount of SNPs: 221,158 “Filtered” and 583,175 “Base”. TER regions contain the following amount of SNPs: 193,941 “Filtered” and 505,877 “Base” datasets. The lines are colored by the position of nucleotide in codons: red – 1^st^, blue – 2^nd^, and green – 3^rd^. Whole-genome fraction of variable positions is 1.3% for the “Filtered” and 4.4% for the “Base” datasets, respectively.

Genomic regions in the vicinity of transcription start (TSS) and termination (TTS) sites are enriched in important regulatory elements^40^,^41^,^42^. The complex formed by transcription factors and RNA polymerase binds to specific conserved sequences within the promoter (Fig. 1). Between the translation and transcription start and, subsequently, termination sites, there are untranslated regions (UTRs) containing conserved elements that regulate translation. Figure 2 shows the distribution of SNPs near the transcription start and end sites. In order to confirm the expected lower sequence variability in the vicinity of TSS and TTS, we extracted 2 kb long fragments centered at TSS and TTS for 20 K high-confidence rice genes (see Methods) and computed the position-specific density of SNPs. At approximately 250 nucleotides upstream of the TSS (see Fig. 2A,C), the SNP density starts to decline, reaching the minimum at the TSS at approximately half of the intergenic value. The steep decline in the SNP rate in the promoter region is in line with the restrictions imposed by the presence of the conserved TFBS in the core promoter [−250, 0] upstream of the TSS (Fig. 3). Within the transcribed region, the density steadily increases from 5′ to 3′ end of the gene and plateaus approximately 300 nucleotides downstream from the TSS as shown in the Fig. 2(A,C). This region of linear increase roughly corresponds to the 5′ UTR. Lower sequence variability in the 5′ UTR regions may be explained by the observation that mutations in this region can change local mRNA structure near the 5′ cap and, therefore, affect translation process. The protein-coding region features fewer variants because of the demand to preserve the amino acid sequence of the product, which is supported by changes mostly in the third codon positions (Fig. 4).

The final stage of transcription is its termination, when the complete transcript dissociates and the RNA polymerase is released from the DNA template. The mechanism of termination is the least understood of the three transcription stages; two competing, yet not fully satisfactory^43^ models known as “allosteric” and “torpedo“^44^ are proposed as possible mechanisms. The distribution of SNPs around the transcription termination site almost mirrors the trend at the TSS, Fig. 2(B,D). SNP density begins to show a decline at about 500 bp upstream from the TTS, reaching the minimum just before the TTS. It then steadily increases for 300 nucleotides downstream from the TTS and then levels off reaching the intergenic level of SNP density. However, the plateau is achieved at over 1000 nucleotides downstream. We suggest that this difference is due to the intrinsic variability of the termination process, with the position of transcription termination being less precisely defined than the transcription start site. This profile of SNP density variation suggests the existence of evolutionary constraints protecting the TTS area, such as requirements to terminate transcription at the appropriate positions^45^, to interact with RNA-binding proteins to regulate mRNA translation^46^, and to accommodate miRNA target sites^47^.

### SNPs in the protein coding regions

Overall, coding regions are more conserved than intergenic regions, promoters and UTRs (Table 1). The frequency of SNPs in introns is 30% higher than in exons, as calculated for the “Filtered” dataset. These results agree with previous observations^2^. There are several interesting trends in SNP distributions near the translation starts and terminations (Fig. 4). First, there is a conserved region immediately upstream of the first codon. The context around the start codon is critical for the translation initiation, as the ribosome may bind at the 5′-most ATG of the mRNA or proceed to the next start codon^48^. Across eukaryotes, the consensus around the start codon is (gcc)gccRccAUGG^49^ (Kozak consensus sequence). In our analysis of 3,000 rice genomes, the consensus is C(G/C)GC(G/C)AUGGCGG, adjusting (but not contradicting) the previously reported consensus for rice (g/c)(A/G)(A/C)(G/C)AUGGC^50^. The initiation of translation (and, therefore, conservation requirements for 5′ UTR) is affected by multiple regulatory mechanisms involving binding of regulatory proteins to specific regions within 5′ UTR, open reading frames and ribosome entry sites^17^.

Translation termination depends on codon-specific release factors that recognize the stop codon sequence in mRNA, as well as on GTP-binding release factors^51^. Efficiency of translation termination depends on the consensus sequence immediately downstream from the stop codon^52^,^53^. Kochetov *et al*.^54^ suggested that nonsense mutations occur significantly more often at the very beginning of 3′-UTR and in the same reading frame as the CDS. They found that *A. thaliana* and *O. sativa* genes with UGA stop codons had more nonsense codons in the first triplet position of 3′ UTR (that may result from weak natural selection for a “reserve” stop signal). Approximately 20–30 nucleotides (nt) upstream of the cleavage-polyadenylation site is the AAUAAA hexamer, which is the consensus signal for the transcript cleavage^17^. Therefore, there are constraints affecting the 3′ UTR sequences manifested as reduced SNP density^55^ (Fig. 4(B,D)). SNPs density gradually increases downstream from the TTS, although the incline of the growth function is smoother than the decline upstream of the first codon. This may be explained by differences in distributions of the 5′ and 3′ UTR lengths (Fig. 2) and weaker constraints on the positional precision of the termination process as compared to transcription initiation.

The third position of the codon, as expected, is more variable than the first two, and that the first one is slightly more variable than the second, which follows from the redundancy of the genetic code and agrees with prior observations in human and mouse^19^.

**Figure 5:**
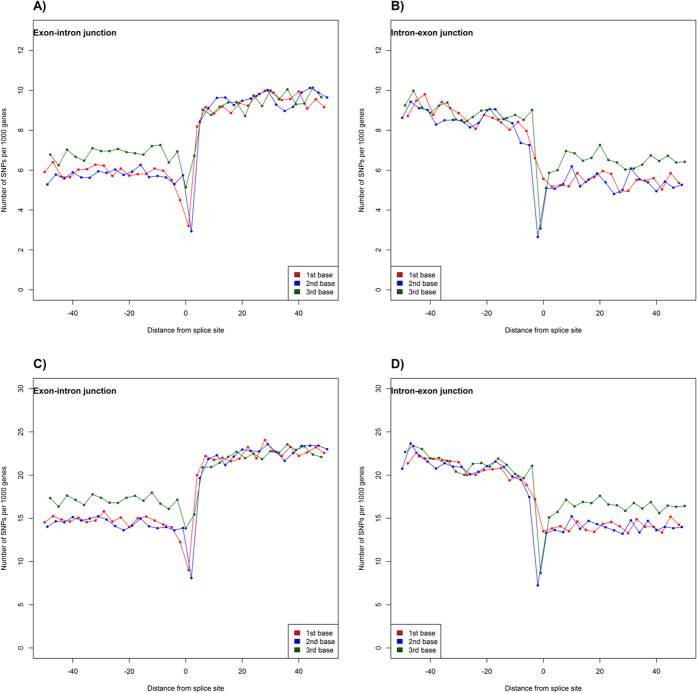
Density of SNP near the exon/intron (**A,C**) and intron/exon (**B,D**) junctions. Panels (**A,B**) correspond to the “Filtered” dataset and panels (**C,D**) to the “Base” dataset. Intron/Exon junctions contain the following amount of SNPs: 40,258 “Filtered” and 99,908 “Base” datasets. Exon/intron junctions contain the following amount of SNPs: 43,138 “Filtered” and 104,797 “Base” datasets. The lines are stratified by position of nucleotide in codons: red – 1^st^, blue – 2^nd^, and green – 3^rd^. Whole-genome fraction of variable positions is 1.3% for the “Filtered” and 4.4% for the “Base” datasets, respectively.

We also analyzed sequence variability in introns (Fig. 5). In both “Filtered” and “Base” datasets, there are more SNPs in introns as compared to the adjacent exons. The exon/intron boundaries are particularly conserved, with the shape of SNP density function around the splicing sites similar to that around the translation and transcription initiation sites and the stop codon.

### Nucleotide composition and SNPs

**Figure 6:**
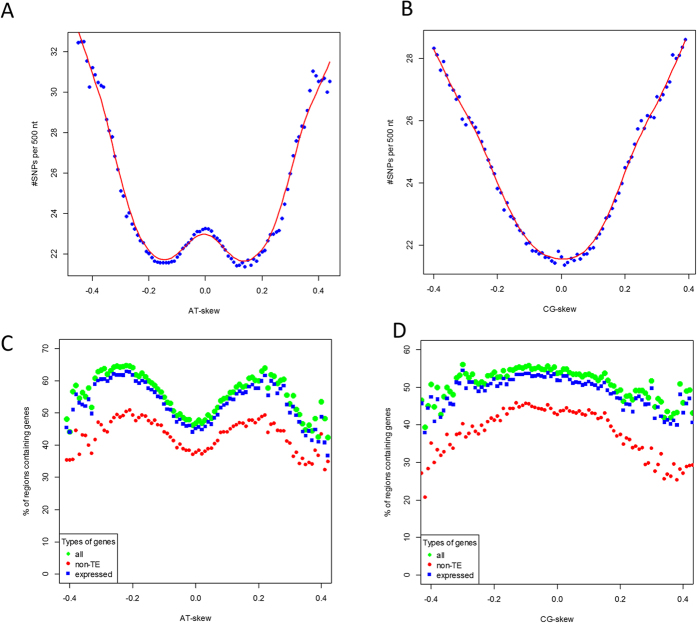
AT and CG skew and the number of SNPs in each 500 nt genomic window. The SNP density of intergenic regions is higher than the SNP density within genes. Coding regions have specific distributions of nucleotide content, resulting in dependence between nucleotide composition and SNP density.

There are two classes of rice genes that differ in function, nucleotide composition, promoter organization, gene expression, and other features^36^,^37^,^56^,^57^,^58^. Here, we investigated distribution of SNPs as a function of the nucleotide composition asymmetry expressed as CG-skew and AT-skew^59^,^60^,^61^,^62^; both dependencies have remarkable shapes (Fig. 6). CG-skew was computed for each 500 nt long genomic region as a difference between the numbers of cytosines and guanines in this region divided by the sum of cytosines and guanines 
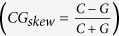
; AT-skew was similarly calculated as 
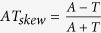
. Nucleotide asymmetry is an important measure, since CG-skew is correlated with the duration a particular DNA region stays in an unpaired, unprotected state during transcription and replication where the longer the duration, the higher the probability of that cytosine transitions to thymine^57^,^59^. Position-specific imbalance between cytosines and guanines was speculated to be related to translational efficiency^63^, avoidance of “kissing interactions,”^64^ DNA methylation^57^, distinct RNA polymerase pause sites in CpG island promoters^65^, and adaptation to extreme environments^37^,^64^,^66^,^67^. In addition, mRNAs tend to be purine-rich (where there is an excess of Gs over Cs)^64^,^68^,^69^. We evaluated the CG skew distribution in rice by dividing the genome into 500 nt long fragments and calculating the nucleotide composition and the number of SNPs in each window (Fig. 6B,D). Since the number of genes in both DNA strands is approximately equal, the plot is symmetric around zero CG-skew. Regions with high absolute values of the CG-skew (>0.3) tend to have high SNP density (Fig. 6B). Genes with high values of the CG-skew are less likely than the genes with low CG-skew to correspond to proteins of unknown function (Cramer’s V = 0.34, see Supplement for details). This observation can be explained by an earlier findings that the peak of CG-skew near the TSS is associated with high transcriptional efficiency^36^,^38^,^59^ and that highly expressed genes have a higher chance to be studied as compared to the genes expressed at a low level. Interestingly, twelve out of top twenty genes by SNP density with |CG-skew|>0.3 are located on chromosomes 11 and 12, which are enriched in disease resistance genes and recent gene duplications^70^. Chromosomes 11 and 12 constitute the most recently evolved part of the rice genome^70^, containing genes that may work differently in different ecotypes of rice subjected to distinct stress conditions present in their respective sources of origin.

Distribution of the AT-skew is shown in Fig. 6(A,C). Variability of the AT-skew was previously linked to tDNA insertion sites^71^, purine loading of mRNA^64^,^68^,^69^, and promoter activity^72^. We found that approximately two thirds of the genes occurring in regions with an absolute value of AT skew above 0.3 correspond to transposable elements (TE), in contrast to genes in genomic regions with AT skew between [−0.3, 0.3], where only 30% of genes correspond to TEs. This effect may also be explained by the observation that protein-encoding regions have distinct patterns of nucleotide composition (manifested as GC content, AT and CG skew patterns)^37^,^59^,^73^ and also have fewer SNPs than the intergenic regions.

### Motifs in promoters associated with SNP density

**Figure 7:**
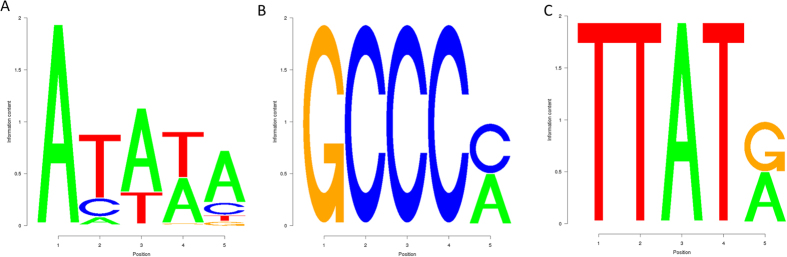
Motifs significantly associated with absence (**A,B**) or presence (**C**) of mutations, located at [−40, −20] for (**A**) and at [−200, −100] for (**B,C**) nt upstream from the TSS. Based on the analysis of 20,367 core promoter regions, Z-scores are: (**A**) −9.95, (**B**) −16.95, and (**C**) 20.6.

In general, DNA sequences in promoters, enhancers and other regulatory elements in eukaryotes are less variable than the rest the genome^74^,^75^,^76^,^77^. However, the patterns of variability may be organism specific and are not well studied in plants. We applied the cisExpress algorithm^78^,^79^ to find regulatory motifs associated with altered SNP density in the region [−250, 50] around the TSS, corresponding to the core promoter and 5′ UTR. Presence of a TATA-box at −30 nt from the TSS and motif GCCC at [−250, −50] are negatively correlated with promoter sequence variability. On the other hand, an (AT)_n_ repeat at 100 or more nucleotides away from the TSS is positively associated with promoter sequence variability (Fig. 7). To estimate statistical significance, we compared the mean number of SNPs in promoters with and without these motifs. The Z-score for the GCCC[ca] motif, which is possibly related to the GCC-box^80^,^81^,^82^ and involved in defense mechanisms, was −16.95, Z-score for the TATA box at −30 was −9.95, and the Z-score for TTAT[ca] was 20.6, suggesting that the GCCC[ca] and TATA-box containing promoters do not tolerate sequence variability, while TTAT[ca]-containing promoters are enriched in SNPs. We suggest that this effect is due to the possible alternative starts of transcription in various ecotypes of *O. sativa* that require a modified promoter to adapt gene expression level to changing environment. To test this hypothesis, one needs to analyze a large collection of 5′-full transcripts from various ecotypes.

### Gene sequence conservation is associated with functionality

Sequence variability in protein-encoding genes may be related to their group-wise functionality, as defined by type-specific biological pathways and processes^83^,^84^. Using the complete 29M set of SNPs, we performed the one sample t-test to find possible associations between DNA conservation in rice genes with GO categories (Table 2). Functional bias was, indeed, pronounced, with the most conserved genes enriched for transcription regulation processes, whereas the least conserved genes encoded membrane located proteins that are involved in stress response and other processes associated with adaptation to the changing environment. GO analysis of the filtered set of SNPs (“Base” dataset) shows similar trends and also indicates that the most conserved genes belonged to the functional category “sequence-specific DNA binding transcription factor” (Supp. Table 5).

Protein family analysis (gene enrichment with PFAM categories) showed that transcription factors feature fewer SNPs than other families. Genes with DUF1618, Myb DNA-binding, zf-C3HC4, and AP2 domains were among the least variable genes. Importantly, we saw the same variability patterns at transcription regulation level. SNP density was the lowest in the promoter regions of the genes belonging to “sequence-specific DNA binding transcription factors”.

## Discussion

Variability of DNA and protein sequences reflects the balance between adaptation and conservation of essential functionality. Analysis of conserved patterns enables “reverse engineering” of function from the sequence data. Active sites of enzymes can be predicted using conserved motifs in protein families, transcription factor binding sites may be identified using conserved elements in homologous promoter sequences, and gene structures can be predicted from conserved fragments of orthologous genes. In general, an unusually high conservation in a DNA or protein sequence implies existence of a biologically important function.

Here, we analyzed the genetic variability within a unique collection of nearly 30M SNPs from the 3,000 Rice Genomes Project^25^. Although the Human 1000 genome project^85^ discovered a larger number of SNPs (80M) from sequencing 2,504 persons, the rice SNP set has a much higher SNP density since the rice genome is almost 10 times smaller than the human genome. To the best of our knowledge, this study has the most precise resolution (one SNP per 20 nucleotides, on average using the “Base” set) of any such study of genome-wide patterns of genic SNP diversity to date.

Here, we demonstrated patterns in genetic variability, established earlier for mammals and observed some novel trends. The rate of polymorphism in introns is known to be higher than in exons, which is consistent with evolutionary constraints in the mutability of protein-coding regions^86^. We confirmed this trend on a subset of common SNPs in rice (MAF>0.01).

We identified promoter motifs associated with gene conservation using the cisExpress program^79^. We observed that TATA+ promoters with TATA-boxes at −30 tend to have slightly fewer SNPs than those without the TATA-boxes. This might be due to the rigidly controlled expression mechanisms of these genes. TATA-box binding proteins recruit other transcription factors into the transcriptosome, with controlled expression profiles needed for genes of more conserved functions. This is consistent with our observation of the differences in distribution of SNPs between various GO classes: gene bodies and promoters of transcription-factor encoding genes have fewer SNPs than other functional classes. At the same time, a number of genes carry “alternative” TATA-boxes 100 or more nucleotides upstream from the usual location, and these regions feature higher SNP densities, perhaps indicating that they are evolving at faster rates. For example, TATA- genes annotated as having “kinase activity” have over 55 SNPs per 250 nt promoter region while TATA+ genes annotated as “sequence-specific DNA binding transcription factor” or “DNA-binding” have three or less. There is also a difference in cellular locations where variable TATA- genes predominate in vacuoles while conserved TATA+ genes occur preferably in cell walls.

In terms of gene families, we have shown that transcription factors feature unusually low SNP density agreeing with their importance of as hubs modulating various downstream cascades. The most genetically variable genes tend to encode membrane-bound proteins, ATPases and serine/threonine protein kinases, key regulators of plant responses to abiotic stresses. Similar trends were highlighted in comparative analysis of human and mouse genomes^87^. The relative conservation of transcription factors and higher genetic variability in the genes responsible for interactions apparently correlates with the need to adjust rapidly changing environmental conditions.

Finally, CG-skew coupled with the associated transcriptional start sites, distributions of regulatory elements, expected lengths of UTR and other trends will be useful to refine gene prediction models in plants, especially the positions of TSSs and TSTs, the boundaries of gene models and the foundation for refinement of genotype to phenotype predictions.

## Materials and Methods

We used a collection of SNPs obtained using the BWA-mem/GATK pipeline from the 3 K rice genome sequence data. SNP calls were made using the BWA-MEM alignment of short reads against the Nipponbare IRGSP-1.0 RefSeq and GATK pipeline. SNPs are available from the SNP-Seek database (http://snp-seek.irri.org)^25^.

### SNP Filtering

We downloaded 29M bi-allelic SNPs called on Nipponbare genome from the SNP-Seek portal. This data was used to generate datasets with varying quality cut-offs.

#### “Base” dataset (16M SNPs)

We excluded SNPs detected in 5 genomes of *Oryza glaberrima,* restricting our analysis of *O. sativa* accessions. Twelve other genomes were excluded due to excessive amounts (i.e., >10,000) of heterozygous singleton SNPs. (see Supplementary Data (Supp. Fig. 1) for distribution of singleton heterozygotes per genome). Next, for each remaining SNP we measured the observed proportion of heterozygotes (H_obs_) and computed the expected proportion of heterozygosity for its allele frequency (H_exp_). The expected ratio H_obs_/H_exp_ for rice is ~0.05, because of high level of inbreeding; however, many SNPs had H_obs_/H_exp_ > 1. SNPs that exhibit high degree of heterozygosity could represent alignment errors due to paralogs that do not occur in the reference genome. We estimated inbreeding coefficients for the 3 К dataset (F_3K_ = 0.9520), *indica* (F_indica_ = 0.9251), and *japonica* (F_japonica_ = 0.9689) as median value of 1 − H_obs_/H_exp_, excluding all SNPs of low frequency (<0.05) and high heterozygosity (H_obs_ > H_exp_). The SNPs, which had more than 2 heterozygotes and violated the condition H_obs_/H_exp_ < 10*(1 − F_3K_), were flagged for removal. The same procedure of flagging was repeated separately for the *indica* and *japonica* subsets, because paralogs are likely to be population specific (e.g. there are SNPs that are nearly 100% heterozygous in *indica* but not in the entire 3000 genomes dataset). All SNPs that have been flagged (in all 3 K samples, in *indica,* and *japonica*) were removed from the 29M dataset, arriving at the “Base” dataset consisting of 16,854,442 SNPs.

#### “Filtered” dataset (5M)

It was obtained by removing SNPs with MAF < 0.01 and maximum per-SNP missing rate 0.1 (PLINK options –maf 0.01 –geno 0.1). “Filtered” dataset contains 4,817,175 SNPs.

### Genomic data and gene selection

The current MSUv7 annotation (http://rice.plantbiology.msu.edu/) of rice contains 55,986 predicted genes and 66,338 gene models^88^. Upon exclusion of pseudogenes, transposable elements, and genes with atypical lengths of 5′ UTR (below 20 nt or above 1000 nt long), we created a high-confidence subset of 20,367 expressed protein-coding genes. Rice genome coverage data were obtained from the Rice 3,000 Genomes project^89^. Genomic regions with zero coverage in all samples were excluded from the analysis. Next, the SNPs were stratified by quality and by genomic regions in terms of their coding, UTR, transcription start and termination, translation start and termination, promoter, etc. For each of the 20,367 genes the number of SNPs per nucleotide was obtained by summing the number of SNPs from the 3000 genomes at this position. A subset of non-synonymous mutations was selected using the SnpEff^87^ tool.

### Gene ontology analysis

Gene Ontology assignments were downloaded from the *Gramene* database (http://ftp.gramene.org/CURRENT_RELEASE/data/ontology/go/go_ensembl_oryza_sativa_japonica.gaf.gz). The map between MSU and RAP-DB locus names was downloaded from the RAP-DB site (http://rapdb.dna.affrc.go.jp/download/archive/RAP-MSU.txt.gz). Enrichment analysis was performed using one-sample t-test implemented in oracle as function *stats_t_test_one*. We excluded GO classes containing less than ten genes.

### Identification of motifs in promoter

Motifs in promoter were found using the cisExpress^79^ tool, which is an improved and enhanced adaptation of an earlier algorithm, Motifer^78^, specifically modified to process large datasets. cisExpress is based an important assumption that function of promoter motifs is position specific. It works in two stages: (1) find ‘seed’ motifs associated with the phenotype of interest, and (2) optimize the ‘seed’ motifs using a genetics algorithm. cisExpress was originally developed to find motifs in promoter associated with gene expression patterns of interest. In this work, we used cisExpress in the ‘off-label’ fashion to find motifs in promoter associated with the SNP density. For each gene, two pieces of information were obtained: sequence of core promoter, defined as region [−250, 50] around the TSS, and number of SNP in this region. Positions of TSS were obtained from the Rice Genome Annotation Project (http://rice.plantbiology.msu.edu/) database and validated using the NPEST algorithm^90^.

### Custom scripts for data analysis

Custom scripts for statistical data analysis and visualization were written in R and C++. In order to calculate the SNP density per genomic region, we wrote an R script that counted the number of SNPs per position for each of the 20,367 selected genes. The number of SNPs was divided by the total number of genes (20, 367), producing the number of SNPs at each position per 1000 genes.

## Additional Information

**How to cite this article**: Tatarinova, T. V. *et al*. Nucleotide diversity analysis highlights functionally important genomic regions. *Sci. Rep.* **6**, 35730; doi: 10.1038/srep35730 (2016).

## Supplementary Material

Supplementary Information

## Figures and Tables

**Table 1:** Fraction of variable nucleotides stratified by genomic regions for the “Filtered” and “Base” datasets.

	Fraction of variable positions per region						
	Intergenic	Promoter	mRNA	CDS	Intron	5′ UTR	3′ UTR
Filtered (5M)	0.0158	0.017	0.0096	0.0070	0.011	0.010	0.011
Base (16M)	0.0517	0.038	0.022	0.016	0.024	0.023	0.024

Whole-genome fraction of variable positions is 1.3% for the “Filtered” and 4.2% for the “Base” datasets, respectively.

**Table 2:** GO terms associated with SNP density.

SNP density	Change from the baseline	log(P-value)	GO ID	Namespace	Name
GO terms for the conserved genes					
5.40%	−19.50%	−50	GO:0006355	Biological process	regulation of transcription, DNA-templated
5.40%	−19.50%	−49	GO:0003677	Molecular function	DNA binding
5.10%	−23.97%	−42	GO:0003700	Molecular function	transcription factor activity, sequence-specific DNA binding
5.10%	−23.97%	−27	GO:0043565	Molecular function	sequence-specific DNA binding
5.20%	−22.48%	−19	GO:0046983	Molecular function	protein dimerization activity
5.60%	−16.52%	−17	GO:0006351	Biological process	transcription, DNA-templated
5.30%	−20.99%	−12	GO:0003682	Molecular function	chromatin binding
5.70%	−15.03%	−12	GO:0003676	Molecular function	nucleic acid binding
6.20%	−7.58%	−11	GO:0005634	Cellular component	nucleus
GO terms for the variable genes					
7.30%	8.82%	−27	GO:0016020	Cellular component	membrane
7.50%	11.80%	−26	GO:0005524	Molecular function	ATP binding
7.30%	8.82%	−24	GO:0005886	Cellular component	plasma membrane
7.30%	8.82%	−18	GO:0000166	Molecular function	nucleotide binding
7.40%	10.31%	−17	GO:0005829	Cellular component	cytosol
7.30%	8.82%	−16	GO:0016021	Cellular component	integral component of membrane
7.50%	11.80%	−15	GO:0016772	Molecular function	transferase activity, transferring phosphorus-containing groups
7.60%	13.29%	−15	GO:0005794	Cellular component	Golgi apparatus
7.70%	14.78%	−15	GO:0004674	molecular function	protein serine/threonine kinase activity
7.50%	11.80%	−14	GO:0004672	Molecular function	protein kinase activity
7.50%	11.80%	−14	GO:0006468	Biological process	protein phosphorylation
7.30%	8.82%	−13	GO:0003824	Molecular function	catalytic activity
7.10%	5.84%	−11	GO:0009507	Cellular component	chloroplast
7.20%	7.33%	−11	GO:0016740	Molecular function	transferase activity
7.40%	10.31%	−11	GO:0016301	Molecular function	kinase activity
7.40%	10.31%	−11	GO:0016310	Biological process	phosphorylation
7.60%	13.29%	−11	GO:0055085	Biological process	transmembrane transport
7.30%	8.82%	−10	GO:0006810	Biological process	transport
7.40%	10.31%	−10	GO:0009570	Cellular component	chloroplast stroma
8.10%	20.75%	−10	GO:0005802	Cellular component	trans-Golgi network
8.40%	25.22%	−10	GO:0006200	Biological process	obsolete ATP catabolic process
8.50%	26.71%	−10	GO:0016887	Molecular function	ATPase activity

Analysis was performed on the 29M dataset; average mRNA mutation density is 6.7%.
