# Supplementary material for: Nucleotide diversity analysis highlights functionally important genomic regions
Source: Sci Rep. 2016 Oct 24;6:35730. doi: 10.1038/srep35730 (PMC5075931; doi:10.1038/srep35730)
Supplement: Supplementary Information [file srep35730-s1.pdf]

Supplementary information for

Nucleotide diversity analysis highlights functionally important genomic regions

Tatiana V. Tatarinova[1,2#], Evgeny Chekalin[3], Yuri Nikolsky[3,4,5], Sergey Bruskin[3], Dmitri Chebotarov[6], Kenneth L. McNally[6], and Nickolai Alexandrov[6#]

1. Center for Personalized Medicine and Spatial Sciences Institute, University of Southern California, Los Angeles, CA, USA
2. Kharkevich Institute for Information Transmission Problems, Russian Academy of Sciences, Moscow, Russian Federation
3. Vavilov Institute of General Genetics, Moscow, Russia
4. F1 Genomics, San Diego, CA, USA
5. School of Systems Biology, George Mason University, VA, USA
6. International Rice Research Institute, Los Baños, Laguna 4031, Philippines

#corresponding authors TT: tatiana.tatarinova@usc.edu; NA: n.alexandrov@irri.org

Genome-wide mutation rates and rare SNP

Understanding the functional significance of polymorphisms is essential for designing better strategies for plant breeding. Modern, cost-effective sequencing and genotyping methods are providing valuable resources for the development of novel approaches. For example, the recent availability of extensive collections of SNPs has allowed researchers to analyze patterns of sequence variability along genomes: bacteria,[1-3] mammals,[4-13] and plants.[14-19]

The original rice SNP dataset contained 29 mil SNPs. We excluded SNPs detected in 5 genomes of *Oryza glaberrima,* restricting our analysis of *O. sativa* accessions. Twelve other genomes were excluded due to excessive amounts (i.e., >10,000) of heterozygous singleton SNPs. Supp. Figure 1 shows distribution of singleton heterozygotes per sample (genome).

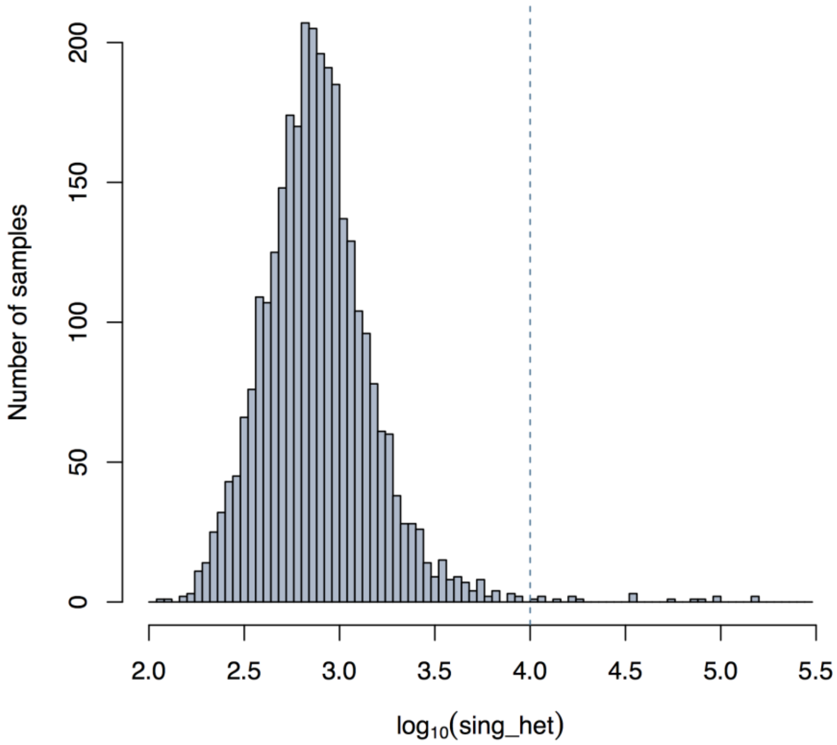

Supp. Figure 1: Distribution of the number of heterozygous singleton SNPs per sample (genome). The vertical dashed line marks a cutoff of 10,000 SNPs separating the seventeen outlier samples.

As an additional measure of quality control for genomes, we computed the number of heterozygous calls that are private to each genome (singleton *hets*) using the Complete SNP set (29M). The distribution of singleton *hets* per genome is skewed to the right, with a long tail. After a log transformation, the distribution becomes bell-shaped (Supp. Figure

1), with peak corresponding to ~670 SNPs per sample. The apparent outliers are separated by the cutoff of 10,000 SNPs, and together contribute 970,976 SNPs (24% of the total singleton *hets*). The high number of singleton *hets* can be due either to high divergence of the sample with respect to the whole collection (five of these outlier accessions are known to be *O*. *glaberrima*) or to contamination. We excluded these samples from further analyses.

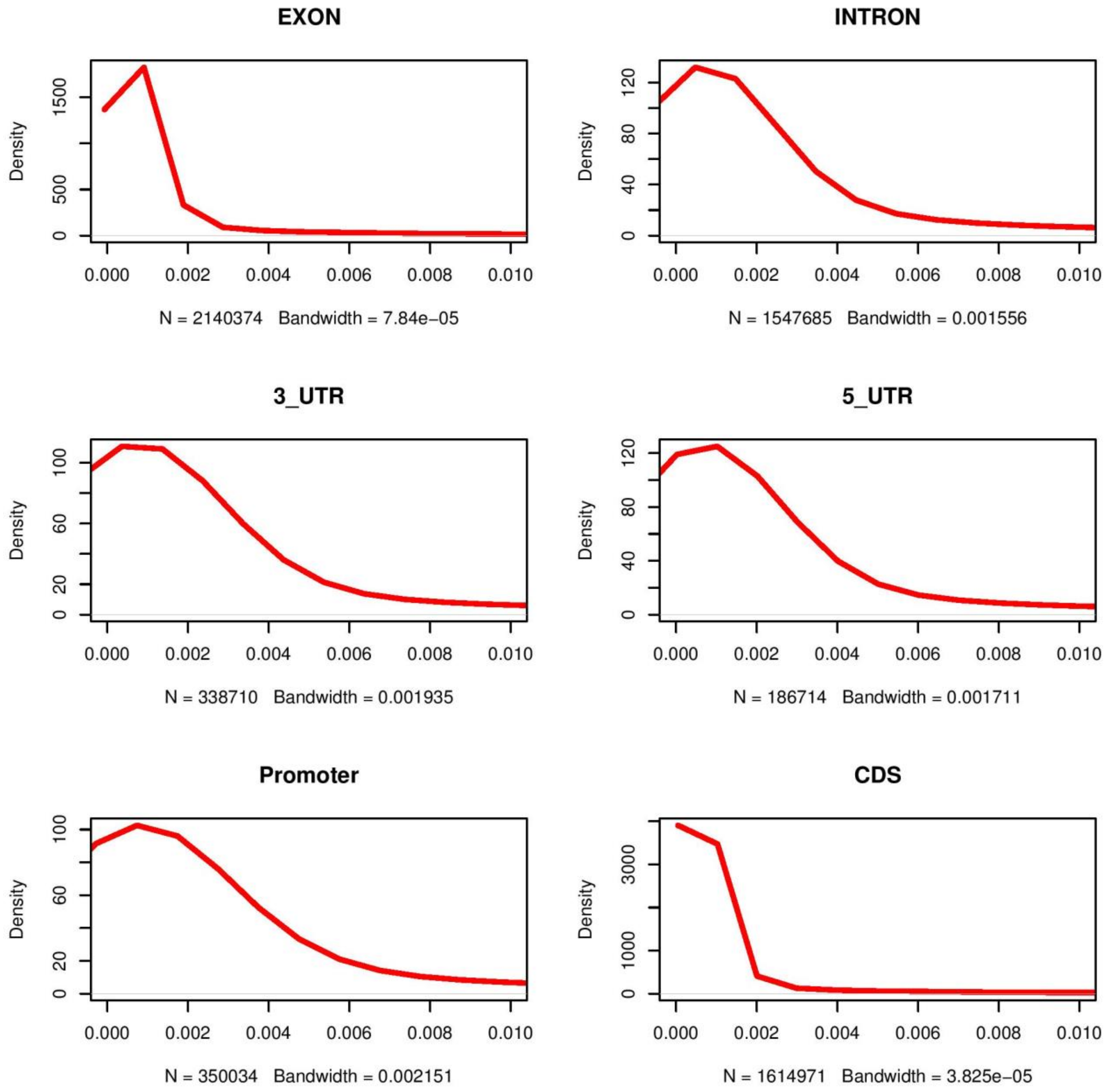

Supp. Figure 2: MAF distribution

Distribution of minor allele frequency (MAF) varies between genomic regions (

Supp. Table 1). Distribution of MAF in the coding regions (CDS and EXON panes in the Supp. Figure 2) are right-skewed (mode shifted towards smaller MAF values). Therefore, with increase of the MAF cut-off, introduced to remove sequencing errors (Supp. Figure 3), increasingly smaller fractions of SNPs in these regions remain in the dataset.

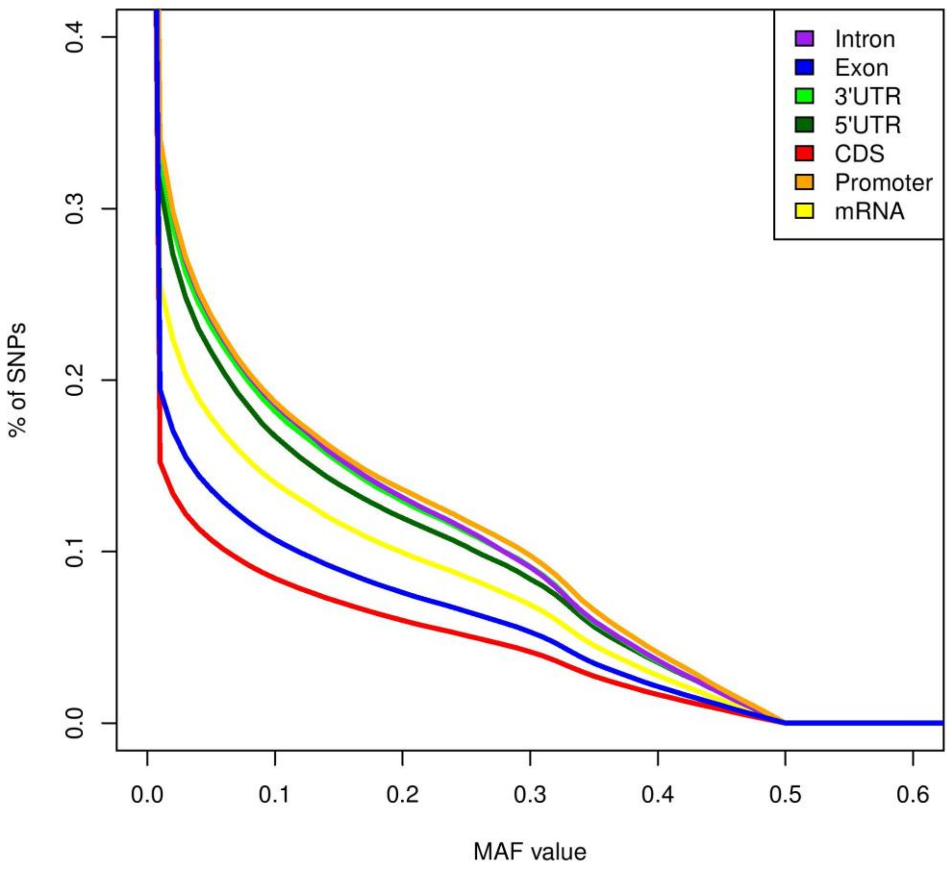

Supp. Figure 3: Percent of SNPs remaining after imposing MAF cut-off

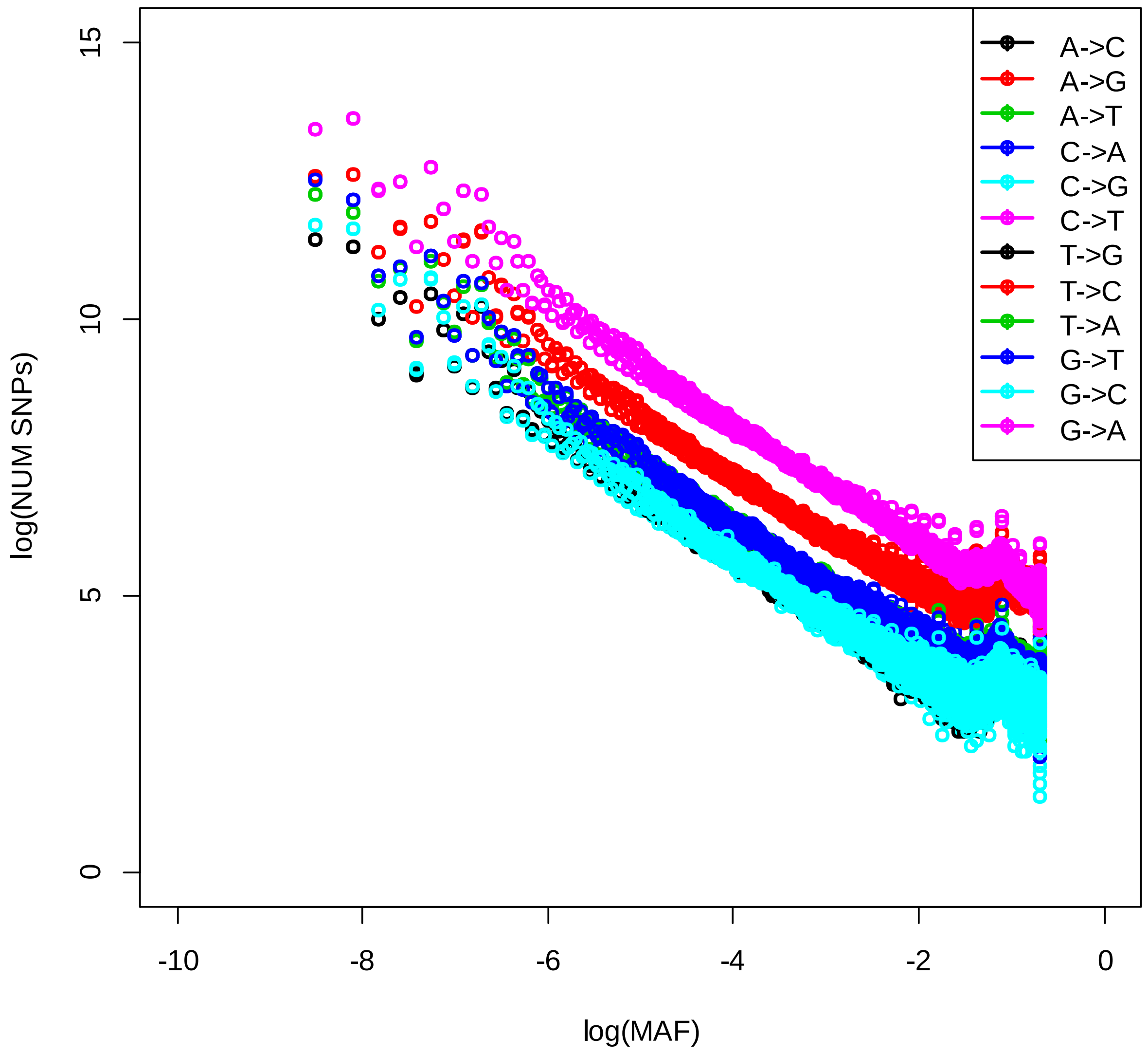

Supp. Figure 4: Number of SNPs as a function of MAF, stratified by the type of substitution, 29M subset.

Supp. Table 1: Summary statistics for MAF in various regions, 29M dataset.

FRAGMENT TYPE	STANDARD DEVIAION	MEAN	MEDIAN
**CDS**	0.086	0.028	4.97E-04
**EXON**	0.096	0.035	4.98E-04
**MRNA**	0.108	0.046	8.28E-04
**WHOLE GENOME**	0.111	0.052	1.70E-03
**5'UTR**	0.117	0.056	1.19E-03
**3'UTR**	0.120	0.060	1.33E-03
**INTRON**	0.120	0.061	1.50E-03
**PROMOTER**	0.124	0.062	1.50E-03

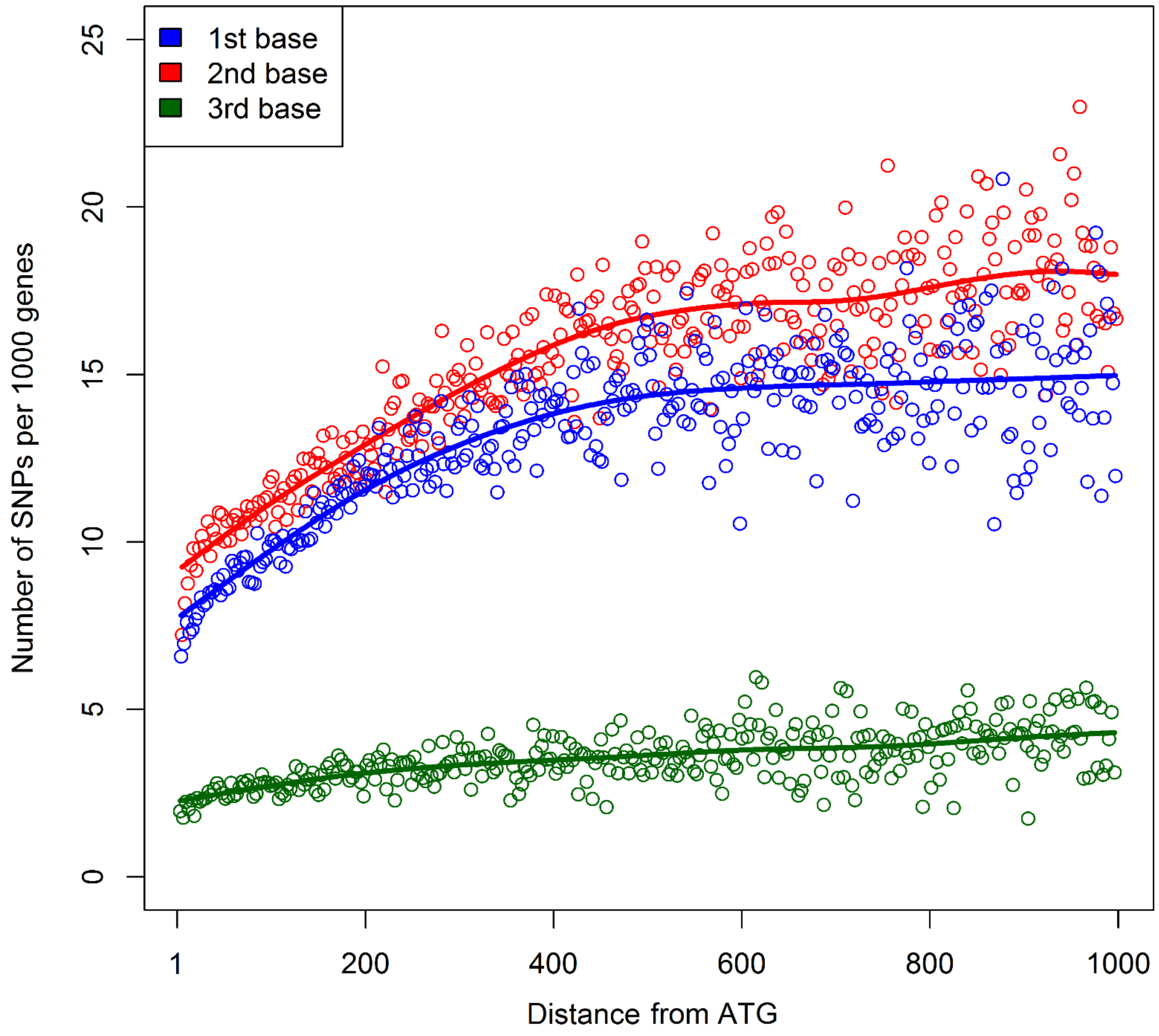

Supp. Figure 5: Distribution of non-synonymous mutations in CDS, 29M dataset.

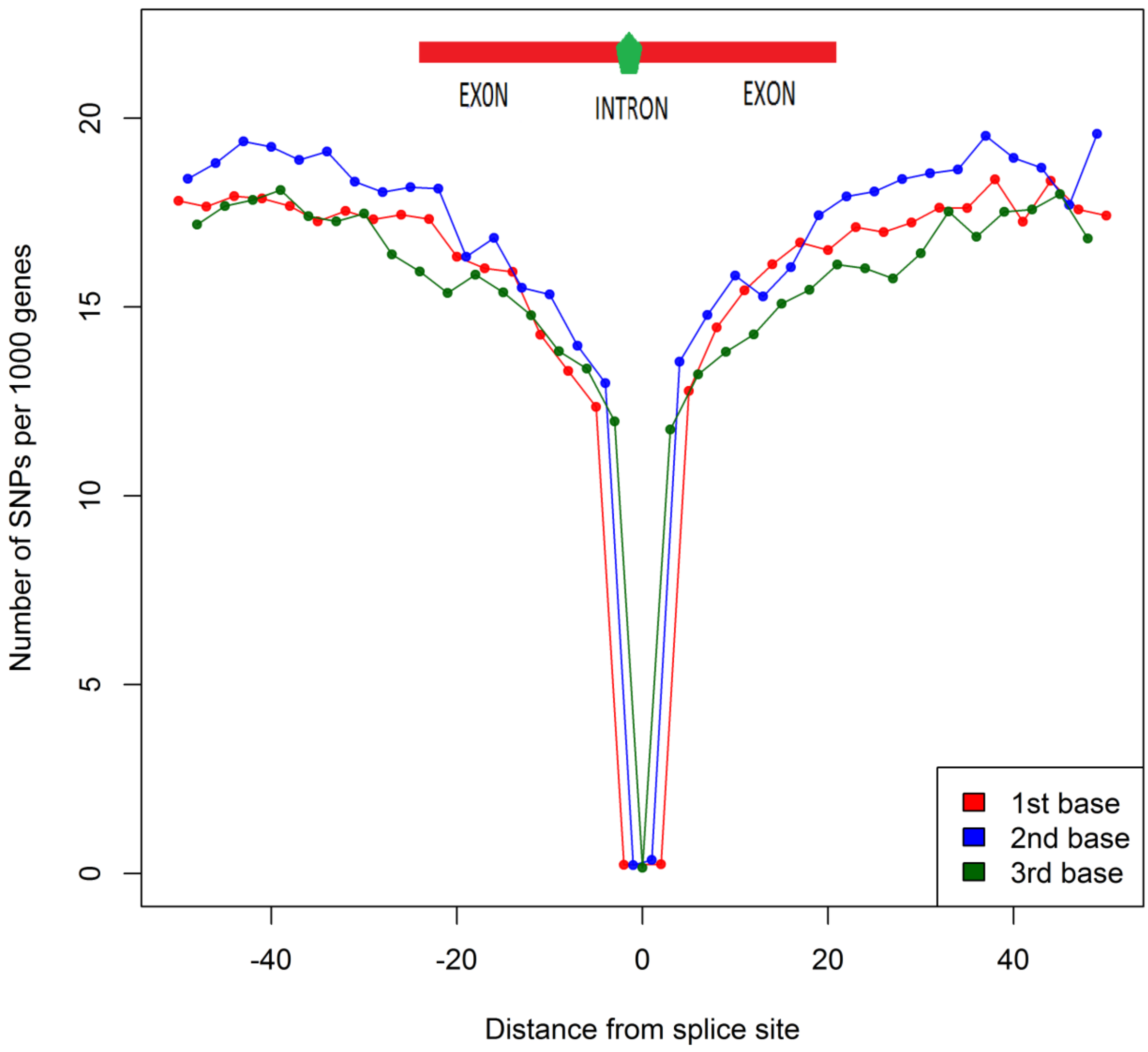

Figure 6: Non-synonymous SNP density for the Exon-Intron-Exon junction, 29M dataset.

When we exclude synonymous positions, the trends are re-arranged (compare Supp. Figure 5 with the main Figure 4). The frequency of non-synonymous SNPs in the 2nd position in the codon is the highest, while the frequency at the 3rd position is the lowest. This effect can also be explained using the codon table: a point mutation in the 3rd position of the codon never results in amino acid change for the 4-fold degenerate cases (such as Proline encoded by codons CCU, CCC, CCA, or CCG), and half of the time for the 2-fold degenerate cases (such as Histidine encoded by CAU or CAC).

PFAM and GO categories

Gene that belong to different GO categories differ in distribution of SNPs (Supp. Figure 7). For example, GO:0006355 "regulation of transcription, DNA-templated" has a higher SNP density as compared to the GO:0006200 category "obsolete ATP catabolic process".

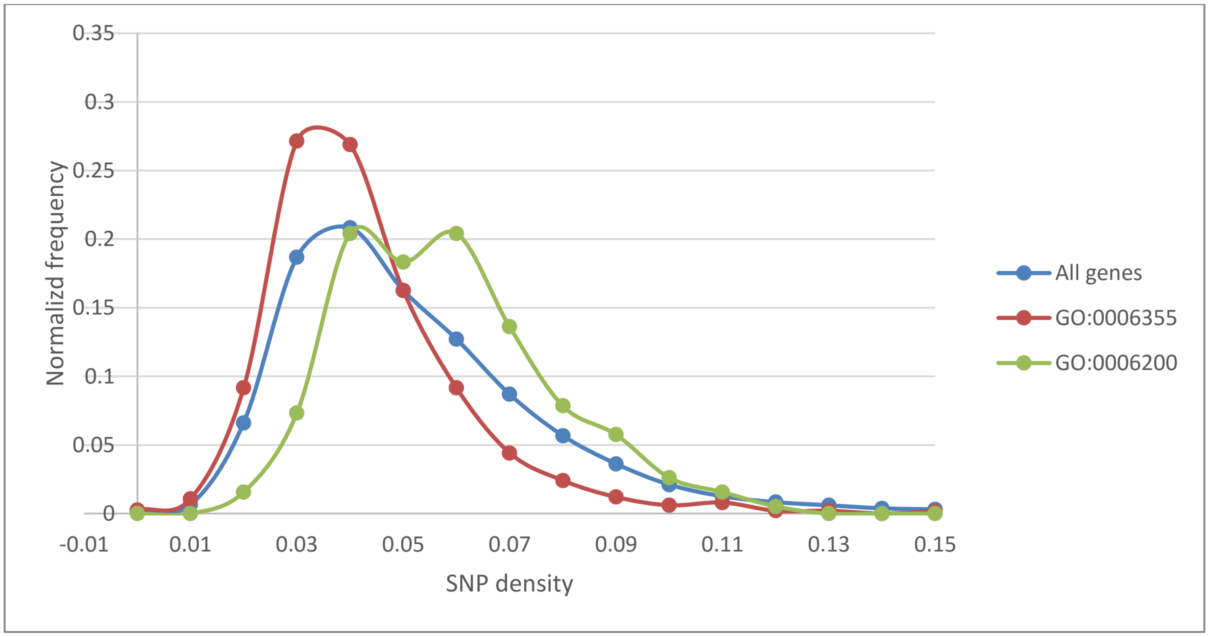

Supp. Figure 7: Comparison of the SNP density distribution for GO:0006355 "regulation of transcription, DNA-templated" and GO:0006200 "obsolete ATP catabolic process" in relation to the SNP density distribution in all genes using the entire 29M dataset.

Next, we divided all rice genes into SNP abundance categories using SNP density in coding and promoter regions. “H” category is defined as 10% of genes ranked by the SNP density, and “L” is the bottom 10% of the genes. GO categories that differ most between the “L” and “H” categories are in the Tables Supp. Table 2 and Supp. Table 5.

Supp. Table 2: GO categories difference

CAT	GO-SLIM	#High	#Low	F(H)	F(L)	Z-score
**F**	sequence-specific DNA binding transcription factor	20	128	1%	10%	-9.61647
**C**	plasma membrane	323	133	24%	12%	8.133084
**F**	DNA binding	40	137	3%	10%	-8.07424
**F**	kinase activity	159	43	11%	3%	7.923264
**P**	carbohydrate metabolic process	101	22	7%	2%	6.738508
**F**	nucleotide binding	211	94	15%	7%	6.40524
**F**	transporter activity	143	50	10%	4%	6.39815
**F**	catalytic activity	300	158	21%	12%	6.382855
**P**	catabolic process	142	51	10%	4%	6.091804
**P**	transport	202	93	14%	7%	5.828575
**F**	hydrolase activity	216	109	15%	8%	5.593938
**P**	response to stress	319	184	**22%**	14%	5.441419
**C**	cell wall	112	37	8%	3%	5.425288
**C**	nucleus	120	178	9%	16%	-4.95284
**F**	transferase activity	150	75	11%	6%	4.624286
**P**	response to abiotic stimulus	191	104	13%	8%	4.472828
**P**	nucleobase, nucleoside, nucleotide and nucleic acid	156	215	11%	16%	-4.3043
**P**	signal transduction	104	48	7%	4%	4.055829
**P**	protein modification process	203	119	14%	9%	4.048254
**C**	Golgi apparatus	36	7	3%	1%	3.984855
**C**	endoplasmic reticulum	59	20	4%	2%	3.804433
**C**	membrane	310	197	23%	17%	3.778593
**C**	cellular component	309	341	23%	30%	-3.66064
**P**	response to biotic stimulus	99	49	7%	4%	3.616344
**C**	extracellular region	66	28	5%	2%	3.276697
**F**	protein binding	285	203	20%	16%	3.166319
**F**	nucleic acid binding	22	45	2%	3%	-3.16517
**F**	receptor activity	18	3	1%	0%	3.112005
**P**	protein metabolic process	93	50	6%	4%	3.09495
**C**	nucleoplasm	5	18	0%	2%	-3.08682
**P**	lipid metabolic process	74	37	5%	3%	3.061764
**P**	cell growth	38	14	3%	1%	3.007337

The category “Sequence-specific DNA binding transcription factor” is 1% in the “H” category and 10% in the “L” category. PFAM category annotation also supports the hypothesis that transcription factors feature fewer SNPs compared to other categories (Supp. Table 3).

Supp. Table 3: PFAM categories difference

CATEGORY	PFAM	#High	#Low	F(H)	F(L)	Z-score
**Family**	DUF1618	0	20	0%	3%	-5.21793
**Domain**	Pkinase	91	23	12%	4%	5.176136
**Family**	LRRNT_2	39	6	5%	1%	4.040701
**Domain**	Myb_DNA-binding	3	17	0%	3%	-3.80892
**Domain**	zf-C3HC4	6	20	1%	3%	-3.51301
**Domain**	AP2	1	11	0%	2%	-3.40984
**Repeat**	LRR_1	60	13	53%	25%	3.375925
**Domain**	ABC_tran	21	2	3%	0%	3.364107
**Repeat**	Ank	10	15	9%	29%	-3.32817
**Family**	Sugar_tr	18	1	2%	0%	3.318228
**Domain**	HLH	0	8	0%	1%	-3.25726
**Domain**	WRKY	0	8	0%	1%	-3.25726
**Domain**	Cu_bind_like	1	10	0%	2%	-3.2125
**Domain**	Lectin_legB	13	0	2%	0%	3.155725
**Domain**	EGF_CA	13	0	2%	0%	3.155725
**Domain**	Glyco_hydro_17	16	1	2%	0%	3.121329
**Domain**	Pkinase_Tyr	31	7	4%	1%	3.120969
**Domain**	F-box	16	30	2%	5%	-3.07749

Distribution of SNPs in the promoter regions is also biased towards functional categories, in a similar fashion (Supp. Table 4).

Supp. Table 4: GO categories and SNP in promoter

CAT	GO description	#High	#Low	F(H)	F(L)	Z-score
**F**	sequence-specific DNA binding transcription factor	60	201	4%	12 %	-8.143
**F**	DNA binding	62	138	4%	8%	-4.71
**P**	nucleobase, nucleoside, nucleotide and nucleic acid	190	304	12%	18 %	-4.27
**C**	cell wall	41	97	3%	6%	-4.12
**P**	biosynthetic process	262	390	17%	23 %	-4.07
**C**	cell	128	216	10%	14 %	-3.79
**P**	anatomical structure morphogenesis	35	74	2%	4%	-3.23
**C**	extracellular region	29	65	2%	4%	-3.15
**F**	transferase activity	144	113	10%	7%	3.00

Supp. Table 5: Analysis of SNPs density per GO category using the 16M dataset

GOSLIM TERM	AVERAGE SNP DENSITY	STDEV SNP DENSITY	NUMBER OF GENES	Z-score	LOG(P-VALUE)	PERCENT INCREASE
**sequence-specific DNA binding transcription factor**	0.018061	0.011489	1196	-11.8581	-32	-18%
**DNA binding**	0.018157	0.012281	1070	-10.2366	-24	-17%
**RNA binding**	0.019057	0.011865	438	-5.19156	-7	-13%
**nucleic acid binding**	0.019267	0.011974	447	-4.82522	-6	-12%
**transporter activity**	0.020665	0.013907	1025	-3.0726	-3	-6%
**structural molecule activity**	0.020066	0.013915	376	-2.69439	-2	-9%
**binding**	0.021292	0.014657	2528	-2.4271	-2	-3%
**hydrolase activity**	0.021191	0.015328	1993	-2.35512	-2	-4%
**signal transducer activity**	0.020084	0.014801	192	-1.79346	-1	-9%
**protein binding**	0.021435	0.017342	2352	-1.58047	-1	-3%
**nuclease activity**	0.019955	0.014866	117	-1.48809	-1	-9%
**enzyme regulator activity**	0.021373	0.014897	199	-0.59373	-1	-3%
**catalytic activity**	0.022055	0.015584	2553	0.177305	0	0%
**lipid binding**	0.022416	0.017119	193	0.337465	0	2%
**transferase activity**	0.022316	0.015799	1271	0.714185	-1	1%
**nucleotide binding**	0.022892	0.019593	1576	1.806708	-1	4%
**carbohydrate binding**	0.025792	0.020204	110	1.968712	-2	17%
**kinase activity**	0.023685	0.020725	1183	2.797077	-3	8%
**oxygen binding**	0.028757	0.016818	175	5.315205	-7	31%

Nucleotide imbalance at the Transcription Start Site and gene expression

TSS has a remarkable feature -peak in AT and CG skews, defined as $AT_{skew} = \frac{\#A-\#T}{\#A+\#T}$, $CG_{skew} = \frac{\#C-\#G}{\#C+\#G}$.[20,21]

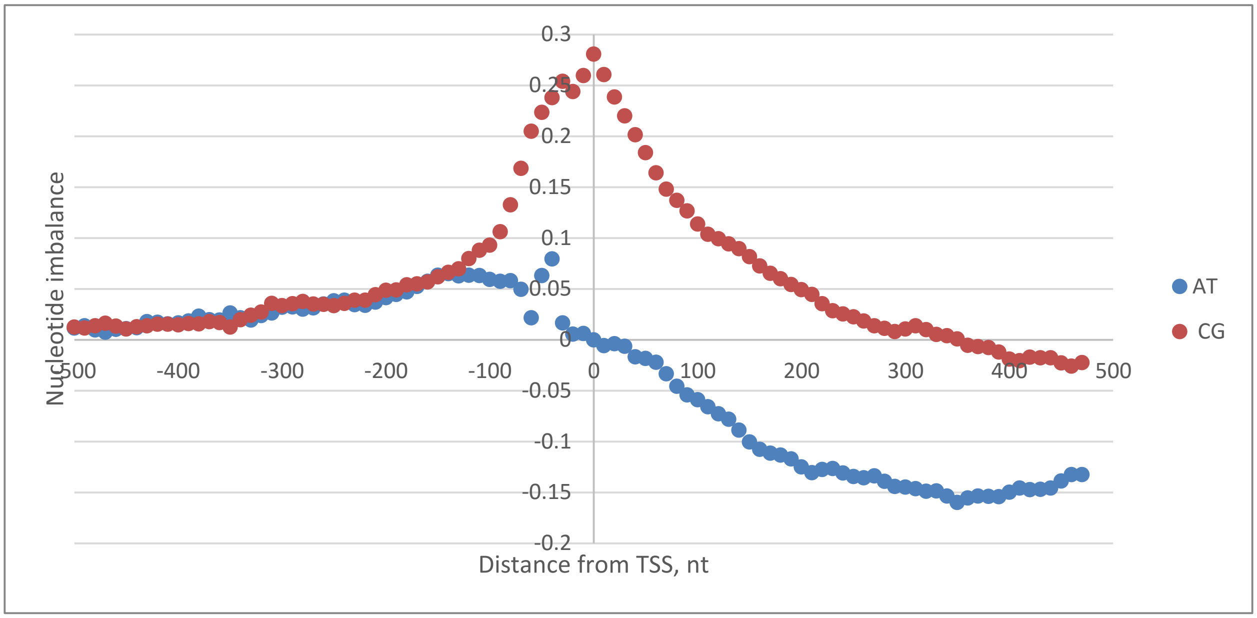

Supp. Figure 8 shows patterns of CG and AT skews in the region [TSS-500, TSS+500] for 20,367 rice genes. AT skew shows a peak associated mostly with the presence of TATA-box at [-40,-20], while CG skew peak is much wider and more pronounced.

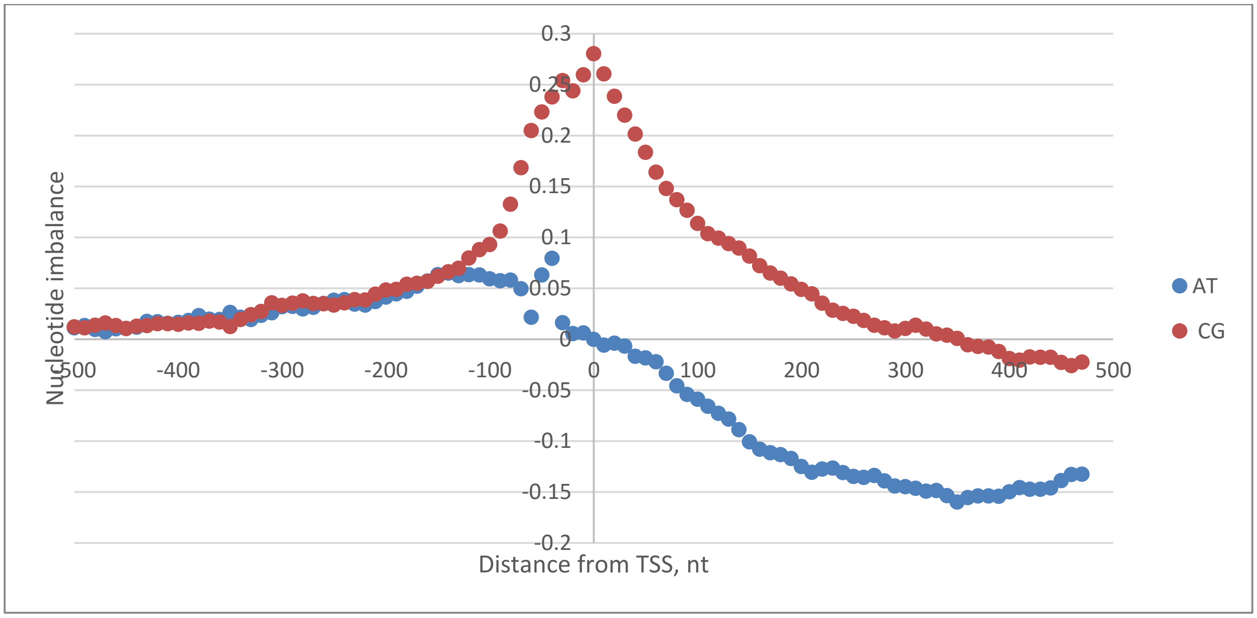

Supp. Figure 8: Nucleotide imbalance at TSS, all genes

Since we assume that the transcription process affects nucleotide imbalance and SNP density in the vicinity of TSS, we created several groupings of genes. First, we ranked the genes by the number of SNPs in the core promoter region [-250,50], sampled 1000 genes from the two tails of the gene list, and computed CG skew profiles. Genes with most mutations in the promoter region (Supp. Figure 9, blue dots) have a higher peak of CG skew compared with the less mutated genes (green dots), and 5'-end of the transcript is more C-rich.

Next, we calculated variability of gene expression (standard deviation of gene expression) in embryo (GSE78997), ranked genes by variability, and computed CG skew profiles for constitutively and differentially (variably) expressed genes. As expected, constitutively expressed genes have a more pronounced peak of CG skew, since they spend more time in the single-strand mode. Comparison of genes in the 1000 SNP-rich list with the 1000 SNP-poor list also shows that SNP-poor genes have 38% increase of variability of gene expression compared to the SNP-rich genes. Variability of gene expression is negatively correlated with the range of CG skew and AT skew (Pearson's correlation coefficients are -0.15 and -0.21, respectively); this observation also supports the claim that constitutively expressed genes, with low variability of gene expression, have more pronounced peaks of nucleotide composition in comparison to differentially expressed genes.

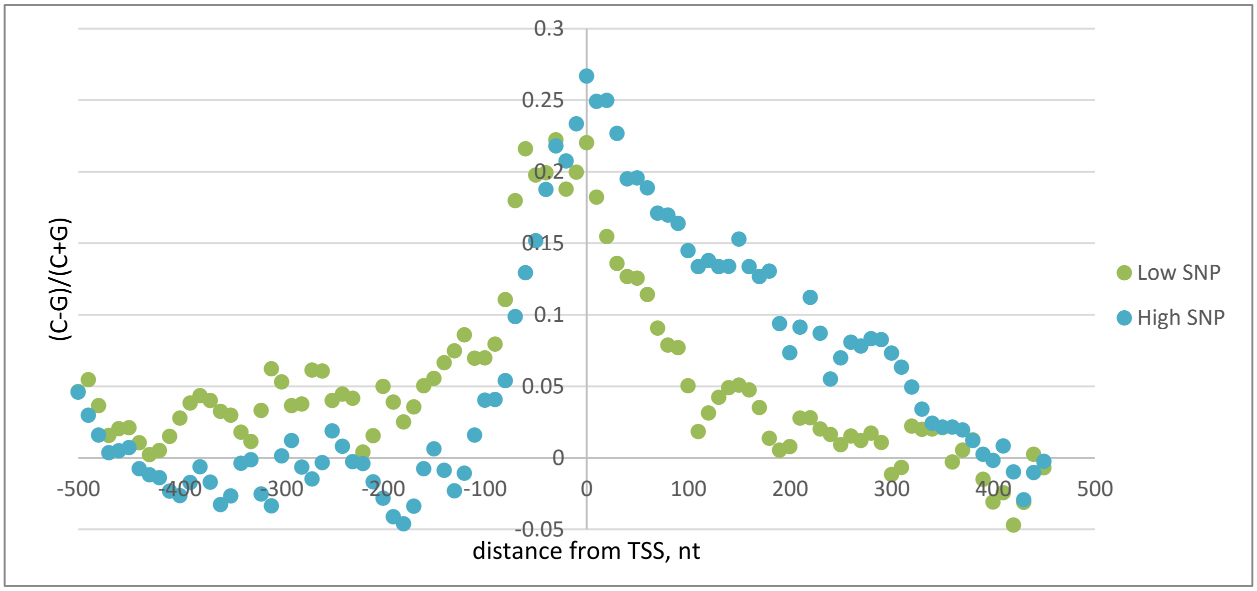

Supp. Figure 9: CG skew for 1000 genes with most and least SNP-rich promoters

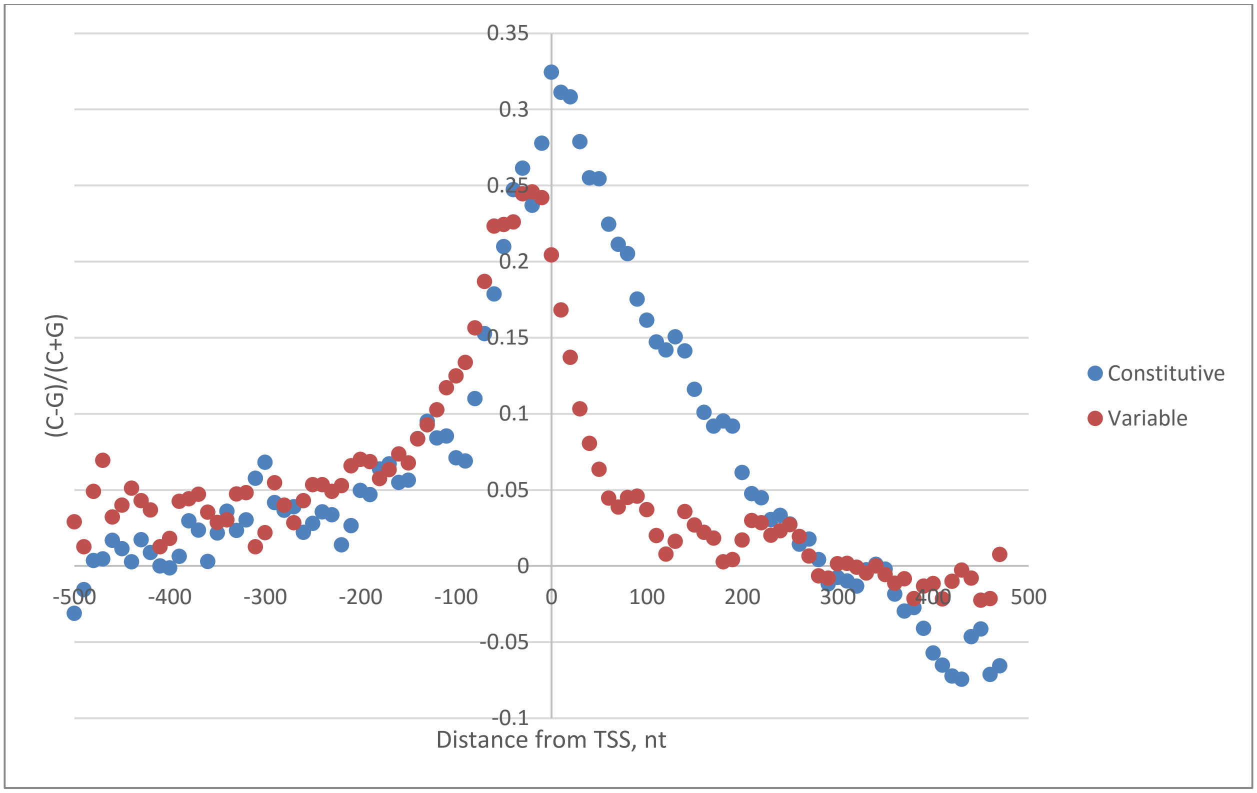

Supp. Figure 10: CG skew for 1000 constitutive and variable genes

Transcription termination models

The final stage of transcription is its termination, when the complete transcript dissociates and the RNA polymerase is released from the DNA template. The mechanism of termination is the least understood of the three transcription stages; two competing, yet not fully satisfactory[22] models known as "allosteric" and "torpedo"[23] are proposed as mechanisms.

In the framework of the allosteric model, transcription termination is caused by the destabilization and/or a conformational change of Pol II EC after transcribing the poly(A) site[24]. However, according to the torpedo model, endonucleolytic cleavage at the poly(A) site creates an entry site for 5′ → 3′ exonuclease, which then degrades the RNA downstream of the cleavage site[24]. Therefore, hybrid models have been proposed, such as allosteric–torpedo and double-torpedo models[25,26]. Modeling of the kinetics of allosteric protein-protein RNA/DNA binding is needed to shed more light on this mechanism.

The profile of SNP density variation suggests the existence of evolutionary constraints protecting the TTS area, such as requirements to terminate transcription at the appropriate positions[27,28], to interact with RNA-binding proteins to regulate mRNA translation[29,30], and to accommodate miRNA target sites.[31,32]

Bibliography

1 Martincorena, I., Seshasayee, A. S. & Luscombe, N. M. Evidence of non-random mutation rates suggests an evolutionary risk management strategy. *Nature* **485**, 95-98, doi:10.1038/nature10995 (2012).

2 Nishida, H. Genome DNA Sequence Variation, Evolution, and Function in Bacteria and Archaea. *Current issues in molecular biology* **15**, 19-24 (2013).

3 Binder, S. *et al.* A high-throughput approach to identify genomic variants of bacterial metabolite producers at the single-cell level. *Genome biology* **13**, R40, doi:10.1186/gb-2012-13-5-r40 (2012).

4 Makova, K. D. & Hardison, R. C. The effects of chromatin organization on variation in mutation rates in the genome. *Nat Rev Genet* **16**, 213-223, doi:10.1038/nrg3890 (2015).

5 Hodgkinson, A. & Eyre-Walker, A. Variation in the mutation rate across mammalian genomes. *Nat Rev Genet* **12**, 756-766, doi:10.1038/nrg3098 (2011).

6 Chuang, J. H. & Li, H. Functional bias and spatial organization of genes in mutational hot and cold regions in the human genome. *PLoS Biol* **2**, E29, doi:10.1371/journal.pbio.0020029 (2004).

7 Varela, M. A. & Amos, W. Heterogeneous distribution of SNPs in the human genome: microsatellites as predictors of nucleotide diversity and divergence. *Genomics* **95**, 151-159, doi:10.1016/j.ygeno.2009.12.003 (2010).

8 Guo, Y. & Jamison, D. C. The distribution of SNPs in human gene regulatory regions. *BMC genomics* **6**, 140, doi:10.1186/1471-2164-6-140 (2005).

9 Fan, W. L. *et al.* Genome-wide patterns of genetic variation in two domestic chickens. *Genome biology and evolution* **5**, 1376-1392, doi:10.1093/gbe/evt097 (2013).

10 Feulner, P. G. *et al.* Genome-wide patterns of standing genetic variation in a marine population of three-spined sticklebacks. *Molecular ecology* **22**, 635-649, doi:10.1111/j.1365-294X.2012.05680.x (2013).

11 Huang, Y., Wright, S. I. & Agrawal, A. F. Genome-wide patterns of genetic variation within and among alternative selective regimes. *PLoS genetics* **10**, e1004527, doi:10.1371/journal.pgen.1004527 (2014).

12 Zhang, X. *et al.* Genome-wide patterns of genetic variation among silkworms. *Molecular genetics and genomics : MGG* **290**, 1575-1587, doi:10.1007/s00438-015-1017-7 (2015).

13 Castle, J. C. SNPs occur in regions with less genomic sequence conservation. *PLoS One* **6**, e20660, doi:10.1371/journal.pone.0020660 (2011).

14 Atwell, S. *et al.* Genome-wide association study of 107 phenotypes in Arabidopsis thaliana inbred lines. *Nature* **465**, 627-631, doi:10.1038/nature08800 (2010).

15 Cao, J. *et al.* Whole-genome sequencing of multiple Arabidopsis thaliana populations. *Nature genetics* **43**, 956-963, doi:10.1038/ng.911 (2011).

16 Weigel, D. & Nordborg, M. Population Genomics for Understanding Adaptation in Wild Plant Species. *Annual review of genetics* **49**, 315-338, doi:10.1146/annurev-genet-120213-092110 (2015).

17 Horton, M. W. *et al.* Genome-wide patterns of genetic variation in worldwide Arabidopsis thaliana accessions from the RegMap panel. *Nature genetics* **44**, 212-216, doi:10.1038/ng.1042 (2012).

18 Lai, J. *et al.* Genome-wide patterns of genetic variation among elite maize inbred lines. *Nature genetics* **42**, 1027-1030, doi:10.1038/ng.684 (2010).

19 Zheng, L. Y. *et al.* Genome-wide patterns of genetic variation in sweet and grain sorghum (Sorghum bicolor). *Genome biology* **12**, R114, doi:10.1186/gb-2011-12-11-r114 (2011).

20 Tatarinova, T., Brover, V., Troukhan, M. & Alexandrov, N. Skew in CG content near the transcription start site in Arabidopsis thaliana. *Bioinformatics* **19 Suppl 1**, i313-314 (2003).

21 Schneeberger, R. G. *et al.* Agrobacterium T-DNA integration in Arabidopsis is correlated with DNA sequence compositions that occur frequently in gene promoter regions. *Functional & integrative genomics* **5**, 240-253, doi:10.1007/s10142-005-0138-1 (2005).

22 Porrua, O. & Libri, D. Transcription termination and the control of the transcriptome: why, where and how to stop. *Nature reviews. Molecular cell biology* **16**, 190-202, doi:10.1038/nrm3943 (2015).

23 Richard, P. & Manley, J. L. Transcription termination by nuclear RNA polymerases. *Genes & development* **23**, 1247-1269, doi:10.1101/gad.1792809 (2009).

24 Rosonina, E., Kaneko, S. & Manley, J. L. Terminating the transcript: breaking up is hard to do. *Genes & development* **20**, 1050-1056, doi:10.1101/gad.1431606 (2006).

25 Luo, W., Johnson, A. W. & Bentley, D. L. The role of Rat1 in coupling mRNA 3'-end processing to transcription termination: implications for a unified allosteric-torpedo model. *Genes & development* **20**, 954-965, doi:10.1101/gad.1409106 (2006).

26 Lemay, J. F. *et al.* The RNA exosome promotes transcription termination of backtracked RNA polymerase II. *Nature structural & molecular biology* **21**, 919-926, doi:10.1038/nsmb.2893 (2014).

27 Yukawa, Y., Sugita, M., Choisne, N., Small, I. & Sugiura, M. The TATA motif, the CAA motif and the poly(T) transcription termination motif are all important for transcription re-initiation on plant tRNA genes. *The Plant journal : for cell and molecular biology* **22**, 439-447 (2000).

28 Tretina, K., Pelle, R. & Silva, J. C. Cis regulatory motifs and antisense transcriptional control in the apicomplexan Theileria parva. *BMC genomics* **17**, 128, doi:10.1186/s12864-016-2444-5 (2016).

29 Szostak, E. & Gebauer, F. Translational control by 3'-UTR-binding proteins. *Briefings in functional genomics* **12**, 58-65, doi:10.1093/bfgp/els056 (2013).

30 Wilkie, G. S., Dickson, K. S. & Gray, N. K. Regulation of mRNA translation by 5'- and 3'-UTR-binding factors. *Trends in biochemical sciences* **28**, 182-188, doi:10.1016/S0968-0004(03)00051-3 (2003).

31 Xie, X. *et al.* Systematic discovery of regulatory motifs in human promoters and 3' UTRs by comparison of several mammals. *Nature* **434**, 338-345, doi:10.1038/nature03441 (2005).

32 Lewis, B. P., Shih, I. H., Jones-Rhoades, M. W., Bartel, D. P. & Burge, C. B. Prediction of mammalian microRNA targets. *Cell* **115**, 787-798 (2003).
